# A Learned Profession

**Published:** 1893-11-18

**Authors:** 


### The Hospital

An Institutional Journal of

**Science, Medicine, Hygiene, Nursing, and News.**

*For Hospitals, Asylums, Infirmaries, Dispensaries, Medical Practitioners, Students, and Nurses.*

Vol. XV.—No. 373.] [Nov. 18, 1893.

### A Learned Profession.

What one finds in the Harveian Oration, delivered by Dr. Pye-Smith a week or two ago is the note of strenuousness; what one misses is the note of wide, luminous, "Baconian" practicality. Exhortation is a good thing; but persuasive light is a better. Dr. Pye-Smith exhorts us to be learned! We are all wearing ourselves out in the effort to attain to more learning. If he had but shown us how to seek medical learning so that everyone of us might forthwith find that which would be most advantageous to our patients, and also to our own mental and moral improvement, what a service he would have rendered to every man throughout the whole profession of medicine, and to every other human being in the community! For it must be admitted that the field of learning which now lies open, even to such a specialiser as the medical man, is so vast that we cannot all cover it in every part. We had better do a little well than attempt too much, and "scamp" most of it.

Pathology is the god of Dr. Pye-Smith's idolatry, and by pathology he means the limited pathology of mere morbid anatomy. Therapeutics, on the other hand, are held up, if not exactly to scorn, at least to a strictly minimised regard. But is not this to affirm the position that the true object of modern medicine is the culture of the physician's mind rather than the restoration or preservation of the common health which the world commits to our care? Is it not the same as saying that the mastery of the mathematics whereby an engineer is enabled to work out his calculations, is of more importance to that engineer than his practical capacity to build, say, a colossal bridge? Whilst the new bridge across the Firth of Forth was in course of erection a well-known mathematician read a paper before a learned society, in which he contended, and, as he thought, proved to demonstration, that the bridge as it was being then constructed never would, could, or should bear the passage across it of a single heavy train. But what is the fact? The practical engineer who built the bridge, notwithstanding the supposed errors in his mathematics, has the satisfaction of looking upon one of the finest pieces of engineering the world has ever seen; and the world on its part may now have the pleasure of riding across the Firth of Forth by the heaviest of trains, and of feeling beneath it a bridge as firm and secure as the solid earth.

Loyalty to medicine demands, at any rate of the man who lives and thrives by his art, loyalty to therapeutics. Moreover, the learning demanded to bring the therapeutic art to anything approaching rational and systematic perfection is not less than that demanded for the acquisition of pathological knowledge and diagnostic capacity. Perhaps it is more. When Dr. Pye-Smith tells us that "treatment without diagnosis brings us unpleasantly near to the charlatan," we are entirely at one with him. But when he asserts that the diagnosis once made "the treatment is in most cases obvious," he is much more rhetorical than scientifically precise. This much it seemed necessary to say, and to say with adequate insistence on behalf of the "practice of medicine" as a daily, honest, and useful "bread-and-butter art."

What is Dr. Pye-Smith's idea of a learned profession? A learned profession he considers to be one which is familiar with all the steps by which its own art has been built up from its foundations in the earliest times; or, to use his own words, "the only true method is the historical one." But if the working medical profession may properly be called upon to accomplish the task of mastering medical history, the task should be well within its powers. Is it within the powers of the average practitioner to acquire a full and intelligent comprehension of medical history? We grant that it ought to be; and we affirm that it would be if medical history were so taught and written that the busy man could find pleasure and advantage in its study. But here are the secrets of the ignorance of medical history which prevails almost universally throughout the profession. Medical history is not taught to medical students; there is no professorship of medical history in any single hospital medical school, nor even at the universities whose boast it is that they teach the liberal arts in a liberal and cultured spirit. On the contrary, the average medical professor or lecturer has nothing but contumely for the past and its history. If he ever refers to it, it is to show how utterly stupid and childish were the men and the methods of former generations, and by contrast how competent and even brilliant are the most mediocre teachers and practitioners of the medical art of the present day. The names of Harvey, Laennec, Malpighi, Hales, Chauveau and Marey, Roy, and Gaskell, quoted by Dr. Pye-Smith, are of course familiar even to the medical student, because they could hardly be otherwise. But every one of them, not excepting Harvey, is really a modern name. Can we, then, call ourselves students of medical history if our studies date no further back than the sixteenth century? Is it possible for students whose researches are confined to a thirtieth part of the whole period of medical history to have a philosophic comprehension of the origin and development of the medical art from the earliest times? The truth is that medicine has produced no historian of the first class. Its Gibbons and its Rankes, its Humes and its Milmans, its Greens and its Motleys are still unknown. To ask the average practitioner of modern times to comprehend his medicine historically is to insist upon his making bricks without straw, and almost, indeed, without clay.

Dr. Pye-Smith makes, and with apparent conviction, the sweeping affirmation that of competent lines of medical study "the only true method is the historical one." We on our part affirm that of all the studies which may properly be called medical the study of medical history is that which is most universally neglected and despised. If Dr. Pye-Smith and ourselves are both "witnesses of truth" then medicine as a learned profession and a practical art must be in a "parlous" state. It is undoubted that from this point of view the medical profession as a whole can by no means be said to be abreast of the general learning of the times. We are all so anxious to be modern that we are nothing but modern. We are in fact not even modern: we are, as an ancient writer said "but of yesterday," and of some of us it may be said, as that same writer proceeded to say, that we "know nothing." For the man who has no knowledge but the knowledge of yesterday and to-day knows, by comparison, "nothing."

It was a pity that Dr. Pye-Smith contented himself with telling his brethren merely of their deficiencies and of the necessity there was for efforts to supply those deficiencies. If he had but been as statesmanlike as he was undoubtedly learned he would have outlined a scheme for the universal teaching of medical history at medical schools. But even if he had not felt competent to produce such a scheme he would at least have so laid the matter upon the consciences of hospital teachers and physicians of competent leisure and learning that they would have felt impelled to set about the task of universal instruction in medical history forthwith. Why, for instance, should not the Harveian Society itself, or still more the Royal College of Physicians, initiate with energy and statesmanlike capacity a scheme for the instruction of all ranks of the profession and of all medical students in the history of medicine? The "historical method," says Pye-Smith, is "the only one"; and his audience appear to have agreed with him. Let them then face the obvious corollary; and let them set to work to give practical effect to so wide-reaching and important a conviction. It is to be feared that real love for our profession and an intelligent determination to raise it as a whole to a constantly heightening level of culture and practical efficiency are exceedingly rare. An appeal lies to all medical men who have leisure, culture, and material resources, to devote themselves with persistent and intelligent loyalty to an object so honourable in itself and so eminently calculated to serve the highest interests of the whole human race.

